# Annotations

**Published:** 1908-08-22

**Authors:** 


### ANNOTATIONS.

### Cosmopolitanism.

While naturally these columns are no place for the discussion of purely political matters, it seems allowable to point out that in a subject which has received a good deal of attention lately from our lay contemporaries there is something of medical interest. Possibly the reports of warlike preparations on the other side of the North Sea may turn out to be like those of the sea-serpent or the giant gooseberry—we do not know; anyhow there must be some sort of virtue in a rallying cry which has succeeded in bringing together some—at first sight, at any rate—queerly assorted and unlikely allies. Our text is taken, however, from the recently published essays of Mr. Frederic Harrison, who is politically unprejudiced, and whose intellectual claims to a hearing are of course strong. His advice is to fraternise with the savant, the poet, the artist of the Fatherland, but to keep our powder dry. The counsel seems excellent. Every medical man who has taken the trouble to examine the sources of the statements in his text-books will be in full sympathy with the cosmopolitanism of science; and in proportion to the extent of such investigations he will be filled with respect for the patient, sagacious labours of the German, who latterly has so out-worked those of other nationalities in medical science. So far as outside knowledge warrants the expression of an opinion, it seems to be the same with many other branches of learning. Nearly everywhere the appeal of the critic is to some *Zeitschrift*, or to ponderous volumes (jocularly called handbooks), published in German cities. The advance of the Western nations, again, is dependent upon scientific research in its widest sense, and therefore upon the teachings of an intellectual oligarchy, to which the German people has shown itself much more heedful than, for instance, our own nation. Altogether it seems likely that if the Teuton should choose to become an active rival, he must prove an exceedingly formidable one.

### The Duties of "Dressers."

There can be few qualified medical practitioners who do not look back now and then with mingled sensations of surprise and regret to the days spent as a surgical dresser at their old hospital: regret for the youthful energy and enthusiasm, the keen pleasure of constantly seeing things new and strange, the light heart and willing hand; surprise that in the hurry and pressure of work the results of distinctly amateur efforts were so good, and that disaster never attended them. Nevertheless, unfortunate incidents do sometimes arise in the best managed hospitals, though their extreme rarity is no mean tribute to the sound common sense of medical students as a class. A recent case at a well-known London hospital illustrates the loophole through which a momentary carelessness may admit the chance of calamity. In this instance a man applied for treatment on account of a swollen leg, he was seen by the house physician, who referred him for treatment to the house surgeon. The latter never saw or heard of the case, but some lotion was ordered by a third year student, who told the man to come next day. In the meantime serious symptoms developed, proved afterwards to be due to acute pancreatitis, and the patient died in the hospital waiting-room early next morning, whither he was wheeled on a barrow. It is satisfactory to note that the lesion of the leg and its treatment had nothing whatever to do with the cause of death; but less so that the hospital rules should be evaded, as admittedly happened. To justify such a breach of the rules by suggesting, as the house physician in his evidence did, that "a student has a certain amount of discretion, having had experience in such cases" does not for one thing represent the situation properly at all, and for another provokes a retort as to what is the value of a qualification and what the use of the regulations of the board. A dresser has not, and ought not to be allowed, any "discretion" at all in treating cases; his duty is to assist his house surgeon, and to carry out instructions, not to diagnose or treat cases, however trivial. If the case we are discussing serves to remind the rising generation of that principle it will have served a most useful purpose.

### School Hygiene.

There are still far too many people about who regard the recommendations of medical officers of health as well-intentioned, but impracticable fads, though this attitude is far less general than it was. To some, therefore, the ideas of Dr. Habgood, of Sutton, as expressed in his paper on the cleansing and disinfection of school buildings, read at the Cardiff Congress of the Royal Sanitary Institute, may sound fantastic; though a careful investigation of the conditions he proposes to remedy would go far to dispel any such conception. The time-honoured slate should be abolished wherever it is still used, says Dr. Habgood, and there is no doubt at all that it is an abomination. Pens and pencils are so frequently sucked, at both ends, that they should be washed daily in a suitable antiseptic. Drinking cups are a fertile channel for the spread of infectious diseases of all kinds, and Dr. Habgood also points out that taps should be so placed that the children cannot apply their lips thereto: he would even prefer that they should drink from the hollows of their often grimy paws, rather than pass on disease from one to another. He advocates also the wearing of stout slippers over unstockinged feet, and the removal of the former before entering the class rooms; the object of this is to encourage more frequent washing, and to avoid chills by sitting with damp boots on, two worthy aims which clash so with the prejudices and customs of the poorer classes that there is not much prospect of their realisation yet awhile. The close association of hats and caps in lobbies is responsible for so free an interchange of parasites that he would like to see the children hatless also, after the manner of the Blue-coat boys. It is pointed out that when pegs are so close that garments hanging on them touch, no amount of cleansing will prevent the spread of undesirable matter. To secure the adoption of radical reforms such as are these, Dr. Habgood will have to educate public opinion a great deal. We trust that in such an effort he will have the undivided support of the medical profession.

